# Multiple Pathogens Contribute to Human Immunodeficiency Virus-Related Sepsis in Addition to *Mycobacterium tuberculosis*: A Prospective Cohort in Tanzania

**DOI:** 10.4103/ijmy.ijmy_80_22

**Published:** 2022

**Authors:** Donatus Bonphace Tsere, Gabriel Mkilema Shirima, Brian S. Grundy, Scott K. Heysell, Stellah G. Mpagama, Shabani Ramadhani Mziray, Peter M. Mbelele

**Affiliations:** 1 Department of medical services, Kibong’oto Infectious Diseases Hospital, Siha, Tanzania,; 2 Department of Global Health and Biomedical Sciences, School of Life Sciences and Bioengineering, Nelson Mandela African Institution of Science and Technology, Arusha, Tanzania,; 3 Division of Infectious Diseases and International Health, University of Virginia, Charlottesville, Virginia, USA; 4 Department of Biochemistry and Molecular Biology, Kilimanjaro Christian Medical University College, Moshi, Kilimanjaro, Tanzania,

**Keywords:** Blood culture, etiologies of sepsis, mortality, people living with human immunodeficiency virus, tuberculosis

## Abstract

**Background:**

Mortality from tuberculosis (TB) sepsis is common among patients living with human immunodeficiency virus (PLHIV). We aimed to detect *M. tuberculosis* (MTB) and additional sepsis etiologies, and mortality determinants in PLHIV.

**Methods:**

This prospective cohort study consented and followed-up PLHIV for 28 days in northern Tanzania. From May through December 2021, patients provided urine and sputum for TB testing in lateral-flow lipoarabinomannan (LF-LAM) and Xpert® MTB/RIF. Bacterial blood culture, cryptococcal antigen, malaria rapid diagnostic, C-reactive-protein (CRP), and international normalized ratio (INR) tests were also performed. Sepsis severity was clinically measured by Karnofsky and modified early warning signs (MEWS) scores. Anti-TB, broad-spectrum antibiotics, and antimalarial and antifungal agents were prescribed in accordance with Tanzania treatment guideline. An independent *t*-test and Chi-square or Fisher’s exact tests compared means and proportions, respectively. *P* < 0.05 was statistically significant.

**Results:**

Among 98 patients, 59 (60.2%) were female. Their mean (standard deviation) age was 44 (12.9) years. TB detection increased from 24 (24.5%) by Xpert® MTB/RIF to 36 (36.7%) when LF-LAM was added. In total, 23 (23.5%) patients had other than TB etiologies of sepsis, including *Staphylococcus aureus*, *Streptococcus pneumoniae, Cryptococcus* spp., and *Plasmodium* spp. Twenty-four (94.4%) of 36 patients with TB had higher CRP (≥10 mg/l) compared to 25 (40.3%) non-TB patients (*P* < 0.001). Nine (9.2%) patients died and almost all had INR ≥1.8 (*n* = 8), Karnofsky score <50% (*n* = 9), MEWS score >6 (*n* = 8), and malnutrition (*n* = 9).

**Conclusions:**

*MTB* and other microbes contributed to sepsis in PLHIV. Adding non-TB tests informed clinical decisions. Mortality was predicted by conventional sepsis and severity scoring, malnutrition, and elevated INR.

## IntroductIon

While human immunodeficiency virus (HIV) infection and mortality have been significantly reduced globally, Eastern and Southern Africa still suffers a disproportionate high burden of people living with HIV (PLHIV) and HIV-related mortality. In 2019, there were 20.7 million PLHIV accounting for 54.4% of the global burden and 300,000 HIV-related deaths which was 43.5% of global morality.^[1–5]^ The use of combined antiretroviral therapy (ART) and antimicrobial prophylaxis has significantly reduced opportunistic infections; however, PLHIV with advanced immune suppression (CD4 + *T* < 200 cells/μL) still develop severe disseminated infection including sepsis.^[6,7]^ The global burden of sepsis is alarming, with an estimated incidence of 31.5 million cases and up to 5.3 million deaths annually. Studies have shown that people with advanced HIV constitute a high proportion of sepsis cases and a high mortality rate as compared to those with HIV-negative status.^[8–11]^ Etiological pathogens of sepsis include bacteria, fungi, parasites, and viruses. As a consequence of altered immune mechanisms in advanced PLHIV, all these microbes may be involved either as a single infection in sepsis or in a polymicrobial pattern.^[12–15]^

Etiological pathogens of sepsis are poorly characterized in sub-Saharan Africa. One contributing factor is a lack of diagnostic tests in most health facilities. Currently, in Tanzania and representative of many regions in sub-Saharan Africa, blood culture, which is the gold standard diagnostic test in sepsis, is only available in tertiary hospitals. In contrast, novel rapid diagnostic tests are more widely adopted for detection of *Mycobacterium tuberculosis* (MTB).^[16–18]^ The use of Xpert® MTB/RIF assay and Mycobacterium Determine^™^ TB lateral-flow urine lipoarabinomannan (LF-LAM) assay has significantly improved TB detection.^[19–24]^ MTB have been recovered in up to 38% of advanced PLHIV with sepsis. Despite diagnosis of tuberculosis (TB) sepsis in people with advanced HIV and initiation of appropriate therapy, mortality has remained substantial.^[8,19–22,25]^ While factors such as delay in treatment initiation can contribute to mortality, polymicrobial infections that may go undiagnosed can also play a part.^[26]^ Hence, for optimization of clinical management of HIV-related sepsis, it is essential to widely characterize etiological agents and those that may not be targeted with current empiric antibacterial approaches. We, therefore, conducted this prospective cohort study in a high TB- and HIV-burdened setting in Tanzania to characterize additional etiological agents of sepsis and factors linked with mortality among PLHIV presenting with features of sepsis.

## Methods

### Study setting

From May through December 2021, the study recruited patients at recruitment sites as well as specimen testing was done in the described laboratory.

### Study design and patients

This prospective cohort study recruited and followed up to determine different etiologies of sepsis and 28-day final treatment outcomes in PLHIV. Patients were recruited if aged 18 years and above, signed a witnessed informed consent, had at least one of the WHO-defined danger signs regardless of CD4+ T cells count, including a body temperature <36°C or ≥39°C, breathing rate > 30 breath/min, heart rate >120 beat/min, unable to walk unaided, had white blood cell count <4000/mm^3^ or > 12000/mm^3^ and or exclusively CD4+ T cell count ≤100 cells/μL. They were excluded if they had hemoglobin ≤5.0 g/dL, obvious localizing wounds, or known bleeding disorder.

Patients with positive urine LF-LAM and/or Xpert MTB/RIF test result for TB received a standard fixed dose comprising rifampicin (150 mg), isoniazid (75 mg), pyrazinamide (400 mg), and ethambutol (275 mg) (RHZE). Patients without TB by any of the test received broad-spectrum antibiotics, antifungal and antimalarial in accordance with standard treatment guideline in Tanzania.^[27]^ As previously described by others described,^[28–30]^ severity of sepsis was clinically measured using modified early warning signs (MEWS) and Karnofsky score. Clinical assessment for either worsening or improvement of danger signs was performed at 3, 5, 10, and 28 days. Patients who recovered and were discharged from the hospital before 28 days were contacted by telephone either directly or through their next of kin.

### Laboratory procedures

#### Specimen collection and transportation

Prior to any medications, the study team collected approximately 30 ml of mid-stream urine and 2-ml sputum for MTB detection by the LF-LAM and Xpert® MTB/RIF assay, respectively, from each patient. A urine catheter was used to collect urine for patients who were unable to void spontaneously.

Blood specimens were collected from all study patients for bacterial culture, complete blood count (CBC), C-reactive protein (CRP), coagulation profile, cryptococcal antigen (CrAg), and CD4+ T count testing. Blood collection was done per existing standard operating procedure at KIDH Laboratory and elsewhere.^[31]^ Briefly, prior to venepuncture, skin was disinfected using 70% with BD isopropyl alcohol swab. In total, 30 ml of blood was drawn whereas 10 ml was dispensed in each of anaerobic and aerobic BACT/ALERT® Blood Culture Media bottles. Of the remaining 10-ml blood samples, 2 ml was dispensed into plain BD vacutainer tubes for CRP and CrAg testing, 4 ml into BD vacutainer ethylenediaminetetraacetic acid (EDTA) tubes for CBC, and 4 ml into BD vacutainer citrate collection tubes plastic for coagulation profile. For patients with CD4 counts done more than 6 months, an additional 4 ml of blood was drawn into BD vacutainer EDTA tubes for CD4 count. Urine LF-LAM was done at point of recruitment and sputum for Xpert® MTB/RIF was processed and tested at Mawenzi, KCMC, and KIDH Laboratories. Blood samples were transported at ambient temperature to KIDH Clinical Laboratory for processing and testing, and results were available within 5 days.

#### Urine tuberculosis Determine^™^ lateral-flow lipoarabinomannan assay

The Determine LF-LAM assay (Alere Inc., Waltham, MA) was performed as per manufacturer’s instruction. In brief, 60 μL of urine sample was applied to specimen site of Determine^™^ LF-LAM Ag test strip and was incubated at room temperature for 25 min. The strip was visually assessed for occurrence of visible patient and control bars. A test was positive for TB if two bar lines for both the patient and control were formed. A test was negative if a line appeared at control bar only.

#### Xpert® Mycobacterium tuberculosis/RIF assay

Xpert® MTB/RIF assay (Cepheid, USA) was performed as described by the manufacturer. Briefly, 1 ml of sputum was mixed with 2 ml of sample reagent (1:2), shaken to homogenize, followed by 15-min incubation at room temperature. Thereafter, 2 mLs of homogenized sputum was transferred into the cartridge and then loaded into the GeneXpert machine for automatic DNA extraction, amplification, and detection of M. tuberculosis complex (MTBC) was reported as previous described.^[23]^

#### Blood culture and identification

Both anaerobic and aerobic blood cultures were performed in BACT/ALERT® 3D blood culture system as per manufactures instructions (bioMérieux, Durham, NC, USA). Briefly, BACT/ALERT® Culture Media bottles were loaded and incubated at 37°C for up to 5 days. Positive blood cultures were detected by a signal in the machine, and it was considered negative if no signal was displayed after 5 days. Identification of positive isolates to species level was performed in accordance with the Clinical and Laboratory Standards Institute guideline,^[32,33]^ and existing standard operating procedure at KIDH Laboratory.

#### Data management and statistical analysis

Data were recorded in a clinical case report form, entered before statistical analysis. The patients were categorized into those with and without TB detected by either Xpert® MTB/RIF or urine LF-LAM. Continuous variables such as age were described as mean standard deviation (SD) and were compared using an independent Student’s *t*-test. Accordingly, proportions were used for categorical variables such as gender, malnutrition measured by body mass index <18.5 kg/m^2^, international normalized ratio (INR) (higher if ≥1.8), and CRP (higher if ≥10 mg/L). A conventional sepsis score for predicting outcome was also measured by MEWS with scores above 6 considered high. Severity of illness was further characterized and categorized as severe at a Karnofsky score <50%. Chi-square or Fisher’s exact test compared proportions among patients with and without TB. A Venn diagram was used to display coinfections among PLHIV. *P* < 0.05 was considered statistically significant. All analyses were performed using the Statistical Package for Social Sciences version 24.0 (IBM SPSS, Armonk, NY, USA).

## Results

### Baseline characteristics of patients with and without tuberculosis sepsis

**Figure 1:**
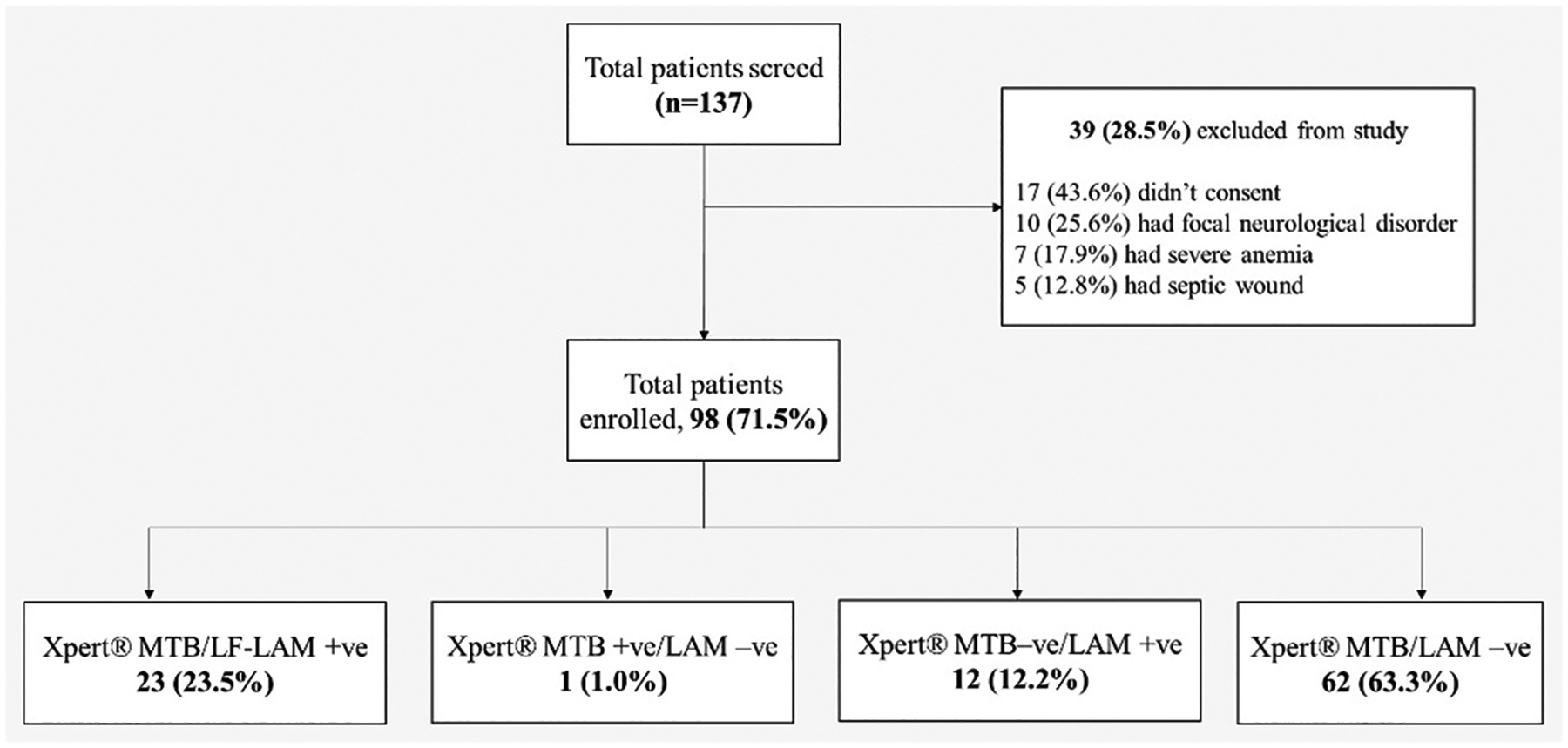
Recruitment procedure patient’s and TB results. TB was diagnosed using Xpert® MTB/RIF in sputum and LF-LAM in urine, TB: Tuberculosis, LF-LAM: Lateral flow lipoarabinomannan, MTB: *Mycobacterium tuberculosis*

Among 137 PLHIV screened, 98 (71.5%) were enrolled [Figure 1]. Of these 98 patients, 39 (39.8%) were male. Their mean age (SD) was 44 (12.9) years. In total, 84 (85.7%) were on ART, 56 (57.1%) had CD4 + T-cells <100 cells/μL and 63 (64.3%) were malnourished. Patients’ sociodemographic and clinical characteristics are shown in Table 1. Overall, patients microbiologically confirmed with TB sepsis were younger than those without TB (mean [SD] age: 39 [12.9] vs. 46 [12.4], *P* = 0.009). Using a MEWS score at 7–8 for sepsis, 16 (25.6%) of patients without TB scored in that higher range compared to 17 (47.2%) with TB (*P* = 0.025). Twenty-seven (32.1%) patients who were on ART had TB compared to 9 (64.3%) patients who were not on ART (*P* = 0.016). Higher CRP at >10 mg/L was also more common in patients with TB compared to those without TB (*P* < 0.001) [Table 1].

### Etiology of sepsis in patients living with human immunodeficiency virus

**Figure 2:**
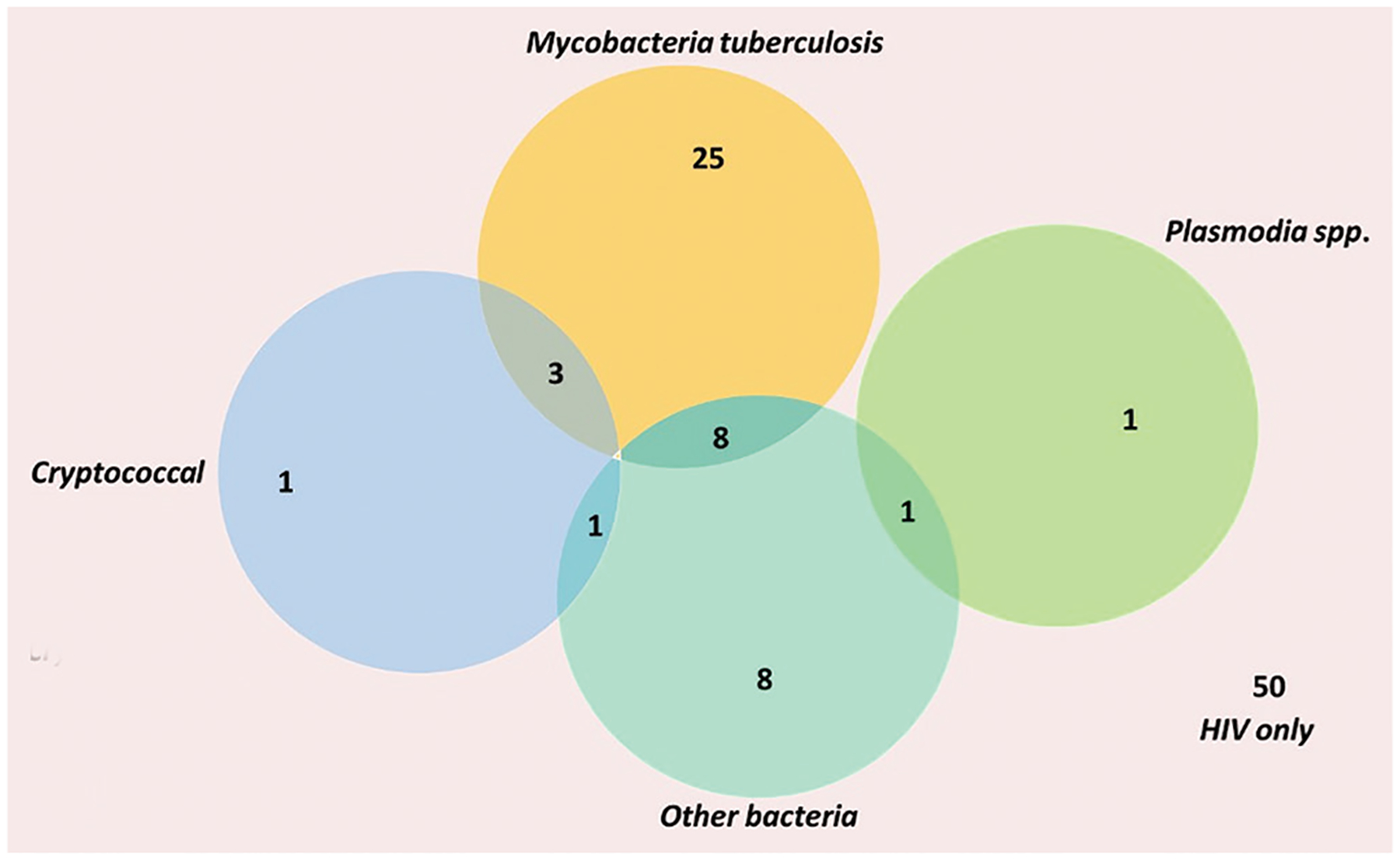
Venn diagram showing different patterns of coinfections in PLHIV, PLHIV: Patients living with human immunodeficiency virus

All 98 PLHIV provided urine for TB-LAM and sputum for Xpert® MTB/RIF testing. In 98 patients, urine LF-LAM increased TB sepsis detection from 24 (24.5%) by Xpert MTB/RIF in sputum sample to 36 (36.7%) by both tests [Figure 1]. Table 2 and Figure 2 show that sepsis in 23 (23.5%) of 98 PLHIV was due to infection by other bacteria (*n* = 18), fungi (*n* = 5), and malaria (*n* = 2). In total, 11 (30.6%) of 36 patients with TB were coinfected by other microbial agents compared to 12 (19.4%) of 62 participants without TB [*P* = 0.207, Table 2]. *Staphylococcus aureus* was the most common cause of sepsis in both patients with (*n* = 4) and without (*n* = 6) TB. Etiological agents for sepsis were not identified in 50 (51.0%) of 98 patients [Figure 2].

### Clinical outcomes

Among 98 patients, 9 (9.2%) died with a mean (SD) age of 30 (7.7), whereas 28-day survivors had a mean age of 45 (12.7) (*P* = 0.001). Mortality was also significantly higher among malnourished compared to those with normal nutrition status (*P* = 0.001), severe form of diseases measured by Karnofsky score below 50% compared to those with score above 50% (*P* = 0.028), high MEWS score (MEWS, 8 [88.9%] vs. 1 [11.1%], *P* = 0.001), and higher INR (8 [88.9%] vs. 1 [11.1%], *P* = 0.025). There was no significant difference in mortality rate among TB patients with/without other pathogens and those without any pathogen detected (HIV alone) (6 [66.7%] vs. 3 [33.3%], *P* = 0.238) [Table 3].

## Discussion

MTB was the primary coinfection and major etiological agent of sepsis among PLHIV in the Kilimanjaro region of Tanzania. MTB is known to have high capacity to evade the immune system and remain dormant within the body awaiting reactivation during conditions of immune suppression, which may account for high TB prevalence.^[34]^ In this study, we observed a prevalence of active TB in more than one-third of patients with advanced HIV disease. Our findings support the high TB prevalence of 38% previously reported in other studies in HIV population.^[13,35,36]^ Apart from TB, coinfection with solitary or multiple pathogens including bacteria like *S. aureus*, fungi like *Cryptococcus* spp., a causative agent for meningitis, and parasites like *Plasmodium* spp., a causative agent for malaria, were commonly detected. This finding is in keeping with a recently reported increase in Gram-positive bacteria recovery among patients presenting with septicemia.^[37]^ The wide spectrum of etiological agents and possible polymicrobial infection in one patient found in our study can partly be attributed to altered cell-mediated and humoral host immunity.^[38,39]^ This diverse cause of sepsis in patients with advanced HIV disease calls for optimal diagnostic algorithm not only to confirm diagnosis but also to guide clinical decisions.

Combination of Xpert® MTB/RIF assay and urine LF-LAM in this study increased TB detection from 24% to 37%. This incremental yield of TB case detection in people living with HIV by both tests was previously reported in the same setting by Byashalira *et al*., in 2019,^[25]^ and in one systematic review and meta-analysis report by Barr *et al*. in 2020.^[19]^ In resource-limited countries like Tanzania, Xpert® MTB/RIF is a frontline diagnostic test for detecting TB in PLHIV.^[40]^ However, Xpert® MTB/RIF requires adequate and quality sputum testing for optimal diagnosis, which is not commonly achieved in immunocompromised, critically ill patients with paucibacillary disease like that reported in the present study. Our findings support the WHO recommendation of using urine LF-LAM for detecting TB among patients with advanced HIV diseases at a CD4+ T-cells <100 cell/μL.^[41]^ In addition, we found a significantly higher CR *P* value in PLHIV with TB coinfections, which is similar to findings from a cohort of Kenyan HIV patients participating in the DREAM program.^[42]^ Our findings and others compliment a systematic review and meta-analysis that recommends CRP as a potential biomarker for screening active TB in high-risk population including PLHIV.^[42,43]^ CRP is a biomarker predominantly released in acute phase following response to interleukin-6 mediated bacterial bloodstream infection such as TB.^[44]^

We found relatively low overall mortality compared to 16.5% previously reported from a similar cohort in the same setting in Tanzania by Byashalira *et al*. in 2019,^[25]^ and in other cohorts such as 21.5% from South Africa and Ghana in 2019,^[45,46]^ and 20% reported from Brazil in 2017.^[47]^ As expected, mortality was high in patients with severe form of sepsis as clinically measured by high MEWS score, a Karnofsky score ≤50%, malnutrition, and coagulopathy biomarker like INR tested.^[48–50]^ Previous studies have argued that early diagnosis and treatment of sepsis limit progression from mild to uncontrolled pro-inflammatory cascades leading to end-organ damage, coagulopathy, clinical deterioration, and ultimately death.^[25,49]^ Certainly, early treatment of active TB confirmed by either Xpert® MTB/RIF or urine LF-LAM tests as well as initiation of empirical broad-spectrum antibiotics for bacterial infection, antifungal therapy for cryptococcal meningitis, and antimalarial in those without TB could partly explain the low mortality in the present study. In addition, low mortality in the current study can also be due to pathogen-guided evidence-based early clinical decision made in support of implementing WHO recommendation to evaluate for worsening or improvement of danger signs at 3, 5, 10, and 28 days.^[51]^ This argument support integration of additional microbiological investigations to optimal recover other causes of sepsis from different sources including but not limited to blood for detecting TB and other bacteria, sputum, and cerebrospinal fluids.^[52]^

The main strength of this study was the holistic application of not only multiple diagnostic tools to confirm the diverse causes of sepsis (TB, bacterial, fungal, and parasitic) in PLHIV from their sputum, urine, and blood but also early initiation of therapy and vigorous monitoring to enforce early clinical decisions. The use of tests other than the TB diagnostic tools to identify bacterial, fungal, and parasitic causes of sepsis addresses prior limitations reported by Byashalira *et al*. in 2019.^[25]^

## Conclusions

MTB in combination with other bacteria, fungi, and parasitic infections were common etiological agents of sepsis in PLHIV. Mortality was predicted by conventional sepsis/severity scoring, malnutrition, and elevated INR. Despite these findings, a comprehensive diagnostic and early treatment and monitoring approach may have led to a relatively lower mortality than previously reported. Our findings support the integration of microbial-diverse diagnostic options in a TB testing algorithm to enhance evidence-based and early clinical decision for optimal outcomes. Ongoing trials such as the randomized clinical trial of early empiric anti-MTB therapy for sepsis in sub-Saharan Africa (ATLAS trial) https://clinicaltrials.gov/ct2/show/NCT04618198 may determine the impact of immediate empiric anti-TB treatment in advanced PLHIV and the optimized composition of antimicrobial regimens for sepsis in clinical settings.

### Limitations of the study

Nonetheless, the study has limitations. First, we were unable to identify etiological agents of sepsis in over half of PLHIV. Finally, this study was done during COVID-19 pandemic, but SARS-CoV-2 was neither presumed to cause sepsis nor tested. Deploying mycobacterial blood culture methods as well as more sensitive multiple pathogen molecular assays such as the RT-qPCR TaqMan® card^[13]^ and metagenomic sequencing technologies would have increased the chance for detecting more putative pathogens.^[53]^

### Ethical clearance

Before taking part in the study, study patients signed a witnessed written informed consent for a protocol approved by the Northern Tanzania Health Research Ethical Committee (reference#: KNCHREC00043/03/2021). Permission to conduct the study was granted by authorities of the KIDH, KCMC, and MRRH. The clinical and laboratory results from this study were used to manage the study patients in the respective health-care setting.

## Figures and Tables

**Table 1:** Sociodemographic and clinical characteristics of people living with human immunodeficiency virus with and without tuberculosis (*n*=98)

Variables	Overall, *n* (%)	Positive TB, *n* (%)	Negative TB, *n* (%)	*P*
Age (years), mean (SD)	44 (12.9)	39 (12.9)	46 (12.4)	0.009*
Gender				
Male	39 (39.8)	14 (38.9)	25 (40.3)	0.975^†^
Female	59 (60.2)	22 (61.1)	37 (59.7)	
Nutritional status, BMI (kg/m^2^)				
<16.0	21 (21.4)	7 (19.4)	7 (11.3)	0.436^†^
16.0–18.4	42 (42.9)	15 (41.7)	27 (43.5)	
18.5–24.9	42 (42.9)	14 (38.9)	28 (45.2)	
Karnofsky score				
<50	57 (58.2)	21 (58.3)	36 (58.1)	0.742^†^
≥50	41 (41.8)	15 (41.7)	27 (41.9)	
MEWS				
<3	1 (1.0)	1 (2.8)	0	0.025^†^
4–6	50 (51.0)	12 (33.3)	37 (59.7)	
7–8	34 (34.7)	17 (47.2)	16 (25.8)	
>8	13 (13.3)	6 (16.7)	9 (14.5)	
Duration of illness (weeks)				
<2	30 (30.6)	6 (16.7)	24 (38.7)	0.032^†^
2–4	46 (46.9)	20 (55.6)	26 (41.9)	
>4	22 (22.4)	10 (27.8)	12 (19.4)	
On ART				
Yes	84 (85.7)	27 (32.1)	57 (67.9)	0.016^†^
No	14 (14.3)	9 (64.3)	5 (35.7)	
Absolute CD4 counts (cells/μL)				
<100	56 (57.1)	21 (58.3)	35 (56.5)	0.520^†^
100–199	29 (29.6)	12 (33.3)	17 (27.4)	
≥200	13 (13.3)	3 (8.3)	10 (16.1)	
Hemoglobin level (g/dL)				
<11.0	31 (31.6)	11 (30.6)	20 (32.3)	0.974^†^
≥11.0	67 (68.4)	25 (69.4)	42 (67.7)	
INR				
<0.8	18 (18.4)	5 (13.9)	13 (21.0)	0.082^†^
0.8–1.1	24 (24.5)	10 (27.8)	14 (22.6)	
>1.1	40 (40.8)	19 (52.8)	22 (35.5)	
CRP (mg/L)				
<10	49 (50.0)	2 (5.6)	47 (75.8)	0.000^†^
10–50	33 (33.7)	19 (52.8)	14 (22.6)	
>50	16 (16.3)	15 (41.7)	1 (16)	

* *P*-value by *t*-test,

† *P*-value by Chi-square or Fisher’s exact test.

ART: Antiretroviral therapy, MEWS: Modified early warning signs, SD: Standard deviation, INR: International normalized ratio, BMI: Body mass index, TB: Tuberculosis, CRP: C-reactive-protein

**Table 2:** Other etiologies of sepsis than *Mycobacterium tuberculosis* in people living with human immunodeficiency virus (*n*=98)

Pathogen isolated	Positive TB patients (*n* = 36), *n* (%)	Negative TB patients (*n* = 62), *n* (%)
No other pathogen	25 (69.4)	50 (80.6)
*Staphylococcus aureus*	4 (11.1)	6 (9.7)
*Streptococcus pneumoniae*	3 (8.3)	2 (3.2)
*Klebsiella pneumoniae*	0	1 (1.6)
*Shigella* spp.	0	1 (1.6)
Coagulase-negative *Staphylococcus*	1 (2.8)	1 (1.6)
*Cryptococcus* spp.	3 (8.3)	2 (3.2)
*Plasmodium* spp.	0	2 (3.2)

TB: Tuberculosis

**Table 3:** Factors associated with mortality among people living with human immunodeficiency virus with sepsis (*n*=98)

Variable	Died (*n* = 9), *n* (%)	Survived (*n* = 89), *n* (%)	*P*
Age (mean)	30 (7.7)	45 (12.7)	0.001*
Gender			
Male	6 (66.7)	33 (37.1)	0.084^†^
Female	3 (33.3)	56 (62.9)	
Nutritional status			
<16	6 (66.7)	8 (9.0)	<0.001^†^
16–18.5	3 (33.3)	39 (43.8)	
>18.5	0	42 (47.2)	
Karnofsky score			
<50	9 (100)	48 (53.9)	0.028^†^
≥50	0	41 (46.1)	
MEWS			
<3	0	1 (11)	<0.001^†^
4–6	1 (2.0)	49 (55.1)	
7–8	2 (5.9)	38 (42.7)	
>8	6 (85.7)	1 (11)	
Duration of illness (weeks)			
<2	0	30 (33.7)	0.08^†^
2–4	7 (77.8)	39 (43.8)	
>4	2 (22.2)	20 (22.5)	
ART use			
No	2 (22.2)	12 (13.5)	0.386^†^
Yes	7 (77.8)	77 (86.5)	
Absolute CD4 count			
<100	5 (55.6)	51 (57.3)	0.357^†^
100–199	4 (44.4)	25 (28.1)	
>200	0	13 (14.6)	
Hemoglobin			
<11	4 (44.4)	27 (30.3)	0.386^†^
>11	5 (55.6)	62 (69.7)	
INR			
<0.8	0	18 (24.3)	0.025^†^
0.8–1.8	1 (4.2)	23 (31.1)	
>1.8	8 (19.5)	33 (44.6)	
Isolated pathogens			
TB with/without other pathogens	5 (55.6)	31 (34.8)	0.513^†^
Other microbes	1 (111)	11 (12.4)	
HIV only	3 (33.3)	47 (52.8)	

* *P*-value by *t*-test,

† *P*-value by Chi-square or Fisher’s exact test.

HIV: Human immunodeficiency virus, ART: Antiretroviral therapy, MEWS: Modified early warning signs, INR: International normalized ratio, TB: Tuberculosis
